# Hourglass Constrictive Neuropathy: A Likely Underdiagnosed Condition with Characteristic Imaging Features

**DOI:** 10.5334/jbsr.3737

**Published:** 2024-09-12

**Authors:** Caroline Chabot, Olivier Barbier, Lokmane Taihi

**Affiliations:** 1 Department of Radiology, Institut de Recherche Expérimentale et Clinique (IREC), Cliniques Universitaires Saint Luc, Université Catholique de Louvain, Brussels, Belgium; 2 Department of Orthopaedic Surgery, Institut de Recherche Expérimentale et Clinique (IREC), Cliniques Universitaires Saint Luc, Université Catholique de Louvain, Brussels, Belgium; 3 Department of Radiology, Institut de Recherche Expérimentale et Clinique (IREC), Cliniques Universitaires Saint Luc, Université Catholique de Louvain, Brussels, Belgium

**Keywords:** Hourglass neuropathy, radial nerve, ultrasound, MRI, neurolysis

## Abstract

*Teaching point:* Hourglass-like constrictive neuropathy should be considered in patients with unexplained peripheral neuropathy symptoms, as imaging may show nerve constriction without evidence of intrinsic or extrinsic compression.

## Clinical History

A 56-year-old male presented with sudden onset of severe pain in his left arm upon awakening, accompanied by significant weakness, particularly in extension of the wrist and fingers. He also experienced hypesthesia over the dorsal surface of his forearm and hand. His medical history included a previous left elbow osteosynthesis following an accident and cervicobrachialgia due to foraminal stenosis at levels C5–C6 and C6–C7, which had resolved spontaneously.

**Figure 1:**
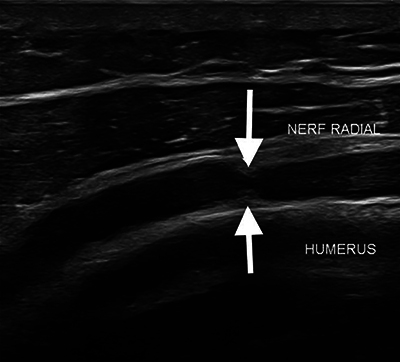
Ultrasound shows focal central narrowing of the radial nerve at the site of its posterior humeral course, with diffuse hypoechoic thickening both upstream and downstream (arrows).

**Figure 2:**
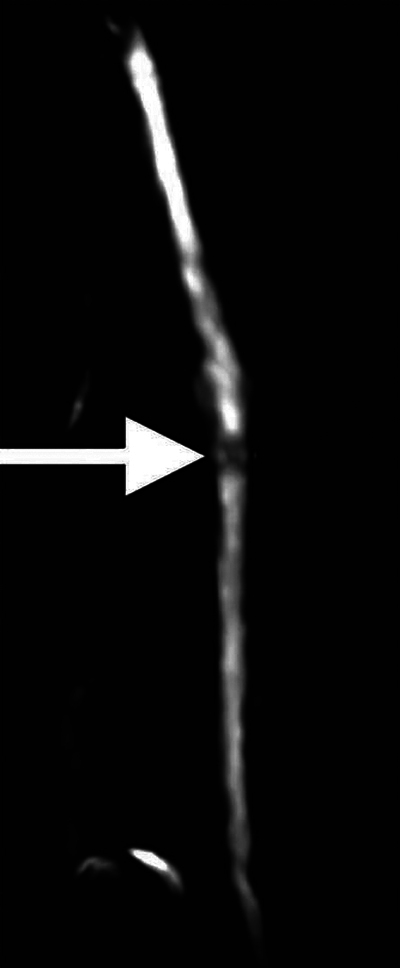
MRI with neurographic 3D STIR sequences shows hyperintense diffuse thickening of the radial nerve with a focal area of narrowing (arrow).

**Figure 3:**
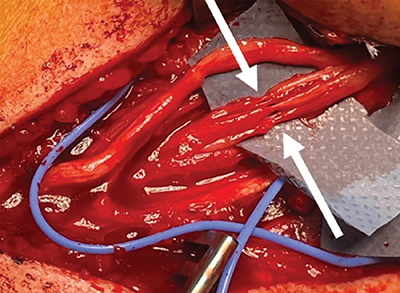
Intraoperative view during surgical neurolysis reveals extreme focal thinning of the radial nerve fascicles, appearing as if constricted by a thread (arrows).

Ultrasound (US) of the affected area showed focal central narrowing of the radial nerve at the site of its posterior humeral course, with diffuse hypoechoic thickening both upstream and downstream (Figure 1, **arrows**). Subsequent magnetic resonance imaging (MRI) with neurographic 3D STIR sequences confirmed these findings, showing hyperintense diffuse thickening of the nerve with a focal area of narrowing (Figure 2, **arrow**). The patient underwent surgical neurolysis of the radial nerve, which revealed extreme focal thinning of the fascicles, appearing as if constricted by a thread (Figure 3, **arrows**). Assessing a torsion mechanism proved difficult.

## Comments

This case highlights a rare observation of hourglass-like constrictive neuropathy, a neurological disorder char-acterized by fascicular constriction of peripheral nerves without intrinsic or extrinsic compression. This condition predominantly affects the motor nerves of the upper extremities and is often associated with Parsonage–Turner syndrome [1].

Patients typically present with severe pain followed by muscle weakness, flaccid paralysis, or limited movement. The exact pathophysiology remains controversial, with proposed mechanisms including initial inflammatory processes, fascicular torsion during motion, local inflammation leading to nerve edema and fascicular fragility, and the development of adhesions between fascicles and adjacent structures.

High-resolution US is instrumental in detecting peripheral nerve constriction and fascicular entanglement, while MRI provides precise localization of nerve involvement. The “bull’s eye sign” on MRI, while useful, has low sensitivity [1].

Treatment has traditionally included exploratory surgery for diagnosis, but there is now a preference for conservative management. Surgery is considered if conservative treatment fails after 3 months or for extensive lesions greater than 2 cm, which may require nerve grafting. Poor prognostic factors include patient age over 50 years, delayed surgical intervention, and severe fascicular thinning [1]. Peripheral nerve transfers can be performed to treat persistent motor and sensory deficits.

## References

[r1] Kim DH, Sung DH, Chang MC. Diagnosis of hourglass-like constriction neuropathy of the radial nerve using high-resolution magnetic resonance neurography: A report of two cases. Diagnostics (Basel). 2020;10(4):232. 10.3390/diagnostics10040232. PMID: ; PMCID: . 32316634 PMC7235890

